# Hospital-mediated realization of enhanced recovery after surgery

**DOI:** 10.3389/fsurg.2025.1554301

**Published:** 2025-05-19

**Authors:** Jia-jing Xu, Xiao-ting Tang, Mao Mao, Qi-ning Yang, Lian-ping Jiang, Wei-cong Fu, Yong-wei Zhou, Xiang-Hong Ye

**Affiliations:** Orthopedic Department, Jinhua Hospital of Zhejiang University, Jinhua Municipal Central Hospital, Jinhua, Zhejiang, China

**Keywords:** enhanced recovery after surgery, hospital management, preoperative fasting, preoperative feeling of hunger, preoperative feeling of thirst

## Abstract

**Purpose:**

Enhanced recovery after surgery (ERAS) has significantly benefited patients and healthcare systems; however, improvements are required, particularly in patient optimization and system implementation. Hospitals, as the main sites for medical activities, can coordinate cooperation among different departments and strategically organize various components of ERAS.

**Methods:**

In March 2023, Jinhua Municipal Central Hospital initiated a comprehensive ERAS program, focusing on fluid and food restriction as the first task assigned. The aim was to reduce the preoperative fasting time throughout the hospital and enhance patients’ preoperative experience. We selected data from the hospital for the first quarter of 2023 as the control group and data from the second and third quarters after the ERAS program implementation as the experimental groups. The collected data included preoperative liquid fasting time (PLFt), preoperative solid fasting time (PSFt), preoperative feeling of hunger (PFoH), and preoperative feeling of thirst (PFoT).

**Results:**

Our study included 24,829 cases. Eight departments included in the statistics revealed a significant reduction in preoperative fasting time, while the reduction of PLFt was more significant. The preoperative feeling of patients was significantly improved, which suggested that the incidence of PFoH and PFoT decreased.

**Conclusions:**

In conclusion, implementing hospital-mediated ERAS is a feasible approach to effectively mobilize resources across departments, coordinate efforts, and address challenges in ERAS implementation. However, the long-term benefits of hospital-mediated ERAS initiatives require comprehensive ERAS protocols and longer follow-up studies for clarity. Additionally, the ERAS procedures need further improvements.

## Background

Enhanced recovery after surgery (ERAS) is a technique with a comprehensive system of thought and practice. ERAS, Initially proposed by Professor Henrik Kehlet of Denmark (1), is designed to reduce perioperative mortality and complication rates through multimodal interventions based on evidence-based medicine. The concept of ERAS has been refined and applied in clinical settings over the past three decades, predominantly in colorectal surgery, and subsequently expanded to various surgical fields (2–7). Employing a patient-centered approach with surgery as the focal point, ERAS integrates multidisciplinary teams, including anesthesia, nursing, nutrition, psychology, and pain management, to optimize perioperative interventions supported by evidence-based practices. The objective is to reduce surgical stress response complications and accelerate patient recovery (8, 9).

ERAS has significantly benefited patients and healthcare systems; however, improvements are required, particularly in patient optimization and system implementation (9). Fundamental components of an ERAS program include the implementation of a multidisciplinary approach essential for the successful execution of ERAS and ensuring the program's sustainability. The core ERAS team typically comprises surgeons, anesthetists, nurses, rehabilitation professionals, and healthcare personnel (10). Effective communication among all disciplines and departments involved in the patient's journey is crucial for the success of ERAS. Unlike other surgical interventions, ERAS transforms perioperative management by enhancing inter-professional communication and fostering continuous commitment from staff and patients to ensure optimal outcomes (11). Collaboration remains a barrier to successful ERAS implementation, with insufficient communication among multidisciplinary team members being a significant challenge (8, 12, 13). Furthermore, a primary challenge in surgical patient care lies in the comprehensive coverage of the patient's healthcare journey by ERAS, which can result in a lack of coordination and holistic planning throughout the process. Therefore, establishing an effective ERAS team is imperative.

In China's later phases of healthcare industrialization, achieving large-scale hospital success was common. Hospitals, as the main sites for medical activities, can coordinate cooperation among different departments and strategically organize various components of ERAS. In contrast to independently implementing ERAS initiatives by individual disciplines, hospitals can ensure effective resource allocation, foster collaboration among professional teams, establish meaningful outcome measures, and evaluate treatment effectiveness through continuous policy development. Our study aimed to explore the potential benefits of hospital-mediated ERAS practices and their realization.

## Methods and materials

This was a retrospective study of existing clinical data approved by the hospital's ethics committee (No. 2023760101). In March 2023, Jinhua Municipal Central Hospital hospital implemented a comprehensive ERAS program, including establishing the hospital's ERAS core team, the extensive promotion of ERAS principles with staff training, and regular ERAS work meetings. In this study, we selected data from the hospital for the first quarters of 2023 (January to March) as the control group and data from the second (April to June) and third quarters (July to September) after the ERAS program implementation as the experimental groups. The study involved the hospital's major surgical departments, including the Department of Gynecology, Department of Orthopedics, Department of Urology, Department of Anus and Intestine Surgery, Department of Hepatobiliary Surgery, Department of ENT, Department of Thyroid and Breast Surgery, and Department of Cardiac and Thoracic Surgery.

Initially, an ERAS core team was established following the institution's operational requirements and the ERAS Society's guidelines (8, 9). The core team comprised the hospital's ERAS leader, liaison group, and department coordinators. The hospital's ERAS leader, appointed by the vice director responsible for relevant affairs, was tasked with developing phased work plans, coordinating internal resources for ERAS implementation, and overseeing collaborative efforts across departments, including outpatient services, inpatient units, operating rooms, and administrative information departments. The ERAS core team, composed of a multidisciplinary team including physicians, nurses, anesthesiologists, and information specialists, was responsible for addressing challenges encountered in the ERAS process, conducting regular ERAS meetings, analyzing data, and providing feedback. The departmental ERAS coordinators were responsible for tracking the progress of specific patients in the ERAS program, monitoring their implementation, and promptly reporting any issues to the ERAS core team for resolution. Surgeons were selected as departmental ERAS coordinators as they are perceived to be more involved in selecting perioperative protocols and implementing specific treatment measures for more patients. Some studies recommend nurses as departmental ERAS coordinators (14, 15), depending on different healthcare systems' operational models and work arrangements.

After the core ERAS team conducted the first meeting, the first task assigned was fluid and food restriction, aiming to reduce the preoperative fasting time throughout the hospital and improve patients' preoperative experience. The American Society of Anesthesiologists recommends the safety and possible benefits of drinking clear liquids containing carbohydrates 2 h before surgery, which can reduce insulin resistance, improve health, and possibly faster recovery (16, 17). After discussing the feasibility of relevant clinical research and hospital implementation during the meeting, the hospital's ERAS leader formulated specific implementation strategies. These strategies include disseminating the latest developments and guidelines on fasting and fluid restriction to improve and unify the awareness and knowledge of healthcare workers. Furthermore, coordination with the nutrition department was established to provide standardized preoperative carbohydrate drinks and solid protein formulations for clinical departments. Additionally, collaboration with the information technology department was made to develop an accessible digital ERAS information retrieval system. This system would facilitate the ERAS team, clinical physicians, and healthcare workers to access patients' fasting and fluid restriction information. Regular meetings of the core ERAS group were scheduled to analyze work data, share experiences, and address challenges encountered. The hospital's ERAS lead coordinated efforts among all departments to resolve any existing issues. Data collected from the fluid and food restriction initiatives primarily included the duration of preoperative fasting, as well as patients' hunger and thirst levels. The respective nurses collected the data upon patients' admission to the operating room and before anesthesia administration. The preoperative liquid fasting time (PLFt) was defined as the time elapsed from the last liquid intake to the start of anesthesia. In contrast, the preoperative solid fasting time (PSFt) was measured from the last solid intake to the initiation of anesthesia. Incidence of the preoperative feeling of hunger (PFoH) and preoperative feeling of thirst (PFoT) in patients were also determined upon patients' admission to the operating room before anesthesia administration by respective nurses.

The mean and standard deviation of all parameters were calculated. Continuous variables were analyzed using the student's *t*-test, while the chi-square test analyzed ordinal and nominal variables. A *p*-value ≤ 0.05 indicated statistical significance. All statistical analyses were performed using the Statistical Package for the Social Sciences software (version 18.0).

## Results

In this study, a total of 24,829 cases were analyzed from January to September 2023. The hospital-wide data from the first quarter (January to March) of 2023 was used as the control group, and data from the second (April to June) and third quarters (July to September) of 2023 were included in the experimental group. There were no significant differences in the basic information of the two groups of patients (Table 1).

**Table 1:** Patient demographics.

Department of gynecology	First quarter	Second quarter	Third quarter	*p*
	(*n* = 757)	(*n* = 900)	(*n* = 1,050)	
Male/Female	757/0	900/0	1,050/0	-
Age	47.58 ± 12.09	49.12 ± 10.25	49.08 ± 13.02	0.786
Department of Orthopedics	(*n* = 1,528)	(*n* = 1,694)	(*n* = 1,846)	
Male/Female	708/820	807/887	899/947	0.392
Age	56.25 ± 9.98	57.45 ± 12.03	57.19 ± 10.71	0.872
Department of Urology	(*n* = 1,125)	(*n* = 1,399)	(*n* = 1,942)	
Male/Female	698/427	856/543	1,228/714	0.475
Age	44.67 ± 9.02	42.19 ± 9.51	44.59 ± 8.59	0.881
Department of Anus & Intestine Surgery	(*n* = 577)	(*n* = 617)	(*n* = 951)	
Male/Female	298/279	317/300	502/449	0.837
Age	52.77 ± 13.56	53.98 ± 15.24	55.02 ± 14.15	0.527
Department of Hepatobiliary Surgery	(*n* = 1,172)	(*n* = 1,424)	(*n* = 1,505)	
Male/Female	621/551	802/622	812/693	0.205
Age	56.29 ± 11.44	55.96 ± 10.21	54.55 ± 9.47	0.544
Department of ENT	(*n* = 800)	(*n* = 909)	(*n* = 1,327)	
Male/Female	408/392	451/458	649/678	0.645
Age	40.58 ± 8.57	42.79 ± 11.98	43.25 ± 13.25	0.742
Department of Thyroid & Breast surgery	(*n* = 440)	(*n* = 567)	(*n* = 895)	
Male/Female	122/318	148/419	204/691	0.204
Age	52.26 ± 11.04	54.18 ± 14.80	53.56 ± 12.47	0.433
Department of Cardiac & Thoracic Surgery	(*n* = 440)	(*n* = 458)	(*n* = 506)	
Male/Female	225/215	232/226	249/257	0.824
Age	50.13 ± 9.57	52.09 ± 9.23	50.98 ± 11.57	0.354

Data are presented as number or Mean ± SD.

The results of preoperative fasting times are presented in Table 2. In the Department of Gynecology, Urology, and Thyroid and Breast Surgery, PSFt and PLFt in the second and third quarters significantly decreased compared to the first quarter (*p* ≤ 0.001). Additionally, PLFt in the Department of Orthopedics and Anus and Intestine Surgery decreased significantly in the second and third quarters (*p* ≤ 0.001), with some improvement in PSFt (*p* ≤ 0.001). In the Department of Hepatobiliary Surgery and ENT, PSFt and PLFt were improved in the second and third quarters (*p* ≤ 0.001). Department of Cardiac and Thoracic Surgery exhibited improvement in PLFt in the second and third quarters (*p* ≤ 0.001), with no significant improvement in PSFt (*p* > 0.05).

**Table 2:** Preoperative fasting time.

Department of gynecology	First quarter	Second quarter	Third quarter	F	*p*
	(*n* = 757)	(*n* = 900)	(*n* = 1,050)		
PSFt	14.95 ± 4.56	12.52 ± 4.87	9.75 ± 3.74	314.97	≤0.001
PLFt	9.19 ± 5.02	6.47 ± 4.83	4.78 ± 4.58	186.15	≤0.001
Department of Orthopedics	(*n* = 1,528)	(*n* = 1,694)	(*n* = 1,846)		
PSFt	14.09 ± 4.80	13.86 ± 4.91	12.97 ± 6.19	20.54	≤0.001
PLFt	10.62 ± 5.51	9.02 ± 5.52	6.74 ± 5.19	221.75	≤0.001
Department of Urology	(*n* = 1,125)	(*n* = 1,399)	(*n* = 1,942)		
PSFt	13.79 ± 5.10	11.40 ± 4.77	9.48 ± 5.83	233.39	≤0.001
PLFt	8.90 ± 5.86	5.60 ± 4.29	4.94 ± 4.81	240.86	≤0.001
Department of Anus & Intestine Surgery	(*n* = 577)	(*n* = 617)	(*n* = 951)		
PSFt	13.96 ± 5.83	15.20 ± 5.30	11.98 ± 11.12	28.42	≤0.001
PLFt	10.62 ± 5.23	7.33 ± 5.19	7.19 ± 9.16	47.20	≤0.001
Department of Hepatobiliary Surgery	(*n* = 1,172)	(*n* = 1,424)	(*n* = 1,505)		
PSFt	13.61 ± 5.09	12.80 ± 4.97	11.50 ± 7.66	40.93	≤0.001
PLFt	8.91 ± 5.36	7.41 ± 5.15	7.11 ± 8.47	27.26	≤0.001
Department of ENT	(*n* = 800)	(*n* = 909)	(*n* = 1,327)		
PSFt	14.27 ± 4.11	13.89 ± 3.91	11.86 ± 5.78	81.58	≤0.001
PLFt	12.51 ± 4.70	12.00 ± 4.41	10.03 ± 5.71	71.99	≤0.001
Department of Thyroid & Breast surgery	(*n* = 440)	(*n* = 567)	(*n* = 895)		
PSFt	14.17 ± 4.98	13.30 ± 5.24	10.70 ± 7.17	57.82	≤0.001
PLFt	10.88 ± 5.61	6.99 ± 4.87	6.25 ± 6.01	104.52	≤0.001
Department of Cardiac & Thoracic Surgery	(*n* = 440)	(*n* = 458)	(*n* = 506)		
PSFt	13.34 ± 4.40	12.84 ± 4.32	12.34 ± 3.84	6.73	0.001
PLFt	9.36 ± 5.19	8.71 ± 5.10	8.93 ± 5.12	1.86	0.155

Data are presented as number or Mean ± SD.

PSFt, preoperative solid fasting time; PLFt, preoperative liquid fasting time.

The statistical results of preoperative feelings of hunger and thirst are provided in Table 3. Patients in the Department of Gynecology, Urology, and Orthopedics exhibited significantly decreased rates of PFoH and PFoT in the second and third quarters compared to the first quarter (*p* ≤ 0.001). Patients in the Department of Thyroid and Breast Surgery had a significantly decreased rate of PFoT in the second and third quarters compared to the first quarter (*p* ≤ 0.001), with a slight decrease in PFoH (*p* ≤ 0.05). Patients in the Department of ENT experienced a decrease in preoperative PFoT in the second and third quarters compared to the first quarter (*p* ≤ 0.05). Patients in the Department of Hepatobiliary Surgery and Anus and Intestine Surgery had a significantly decreased rate of PFoT in the second and third quarters compared to the first quarter (*p* ≤ 0.001); however, there was no significant improvement in PFoH (*p* > 0.05). Patients in the Department of Cardiac and Thoracic Surgery exhibited no significant change in PFoH and PFoT between the second and third quarters compared to the first quarter (*p* > 0.05).

**Table 3:** Preoperative feeling of hunger and thirst.

Department of gynecology	First quarter	Second quarter	Third quarter	*p*
	(*n* = 757)	(*n* = 900)	(*n* = 1,050)	
PFoH	452 (59.7%)	351 (39.0%)	148 (14.1%)	≤0.001
PFoT	438 (57.9%)	267 (29.7%)	109 (10.4%)	≤0.001
Department of Orthopedics	(*n* = 1,528)	(*n* = 1,694)	(*n* = 1,846)	
PFoH	899 (58.8%)	860 (50.8%)	652 (35.3%)	≤0.001
PFoT	785 (51.4%)	655 (38.7%)	299 (16.2%)	≤0.001
Department of Urology	(*n* = 1,125)	(*n* = 1,399)	(*n* = 1,942)	
PFoH	518 (46.0%)	455 (32.5%)	225 (11.6%)	≤0.001
PFoT	389 (34.6%)	342 (24.4%)	186 (9.6%)	≤0.001
Department of Anus & Intestine Surgery	(*n* = 577)	(*n* = 617)	(*n* = 951)	
PFoH	290 (50.3%)	325 (52.7%)	451 (47.7%)	0.121
PFoT	244 (42.3%)	208 (33.7%)	176 (18.5%)	≤0.001
Department of Hepatobiliary Surgery	(*n* = 1,172)	(*n* = 1,424)	(*n* = 1,505)	
PFoH	588 (50.2%)	701 (49.2%)	734 (48.8%)	0.769
PFoT	430 (36.7%)	408 (28.7%)	395 (26.2%)	≤0.001
Department of ENT	(*n* = 800)	(*n* = 909)	(*n* = 1,327)	
PFoH	432 (54.0%)	440 (48.4%)	621 (46.8%)	0.005
PFoT	395 (49.4%)	433 (47.6%)	578 (43.6%)	0.021
Department of Thyroid & Breast surgery	(*n* = 440)	(*n* = 567)	(*n* = 895)	
PFoH	238 (54.1%)	280 (49.4%)	405 (45.3%)	0.009
PFoT	195 (44.3%)	158 (27.9%)	162 (18.1%)	≤0.001
Department of Cardiac & Thoracic Surgery	(*n* = 440)	(*n* = 458)	(*n* = 506)	
PFoH	225 (51.1%)	232 (50.7%)	249 (49.2%)	0.824
PFoT	200(45.5%)	198(43.2%)	209(41.3%)	0.199

PFoH, preoperative feeling of hunger; PFoT, preoperative feeling of thirst.

Figures 1–8 depict the changing trends in the PSFt and PLFt for patients in different departments and the changing trends in the PFoH and PFoT among patients in each department.

**Figure 1:**
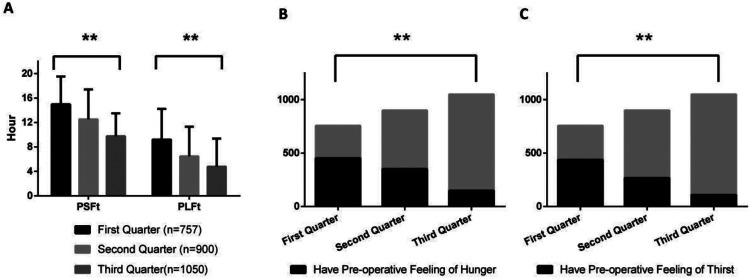
Department of gynecology, **(A)** preoperative liquid fasting time (PLFt) and of preoperative solid fasting time (PSFt) of patients, **(B)** preoperative feeling of hunger (PFoH) of patients, **(C)** preoperative feeling of thirst (PFoT) of patients. **p ≤ 0.001.

**Figure 2:**
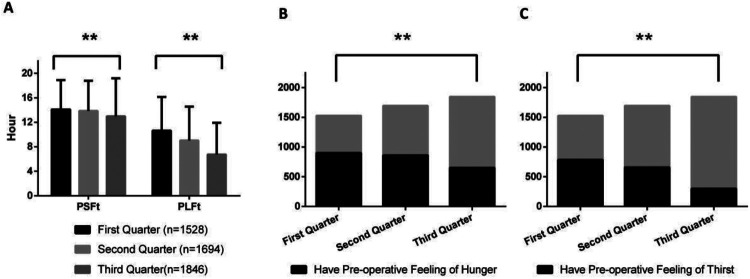
Department of orthopedics, **(A)** preoperative liquid fasting time (PLFt) and of preoperative solid fasting time (PSFt) of patients, **(B)** preoperative feeling of hunger (PFoH) of patients, **(C)** preoperative feeling of thirst (PFoT) of patients. **p ≤ 0.001.

**Figure 3:**
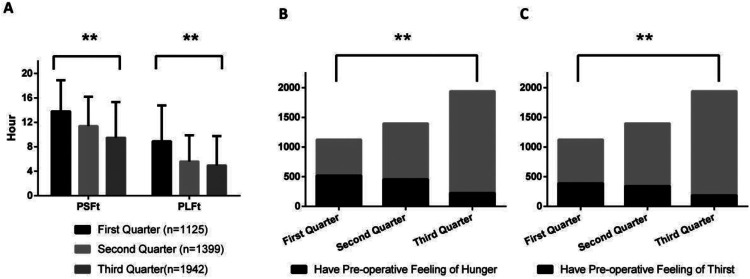
Department of urology, **(A)** preoperative liquid fasting time (PLFt) and of preoperative solid fasting time (PSFt) of patients, **(B)** preoperative feeling of hunger (PFoH) of patients, **(C)** preoperative feeling of thirst (PFoT) of patients. **p ≤ 0.001.

**Figure 4:**
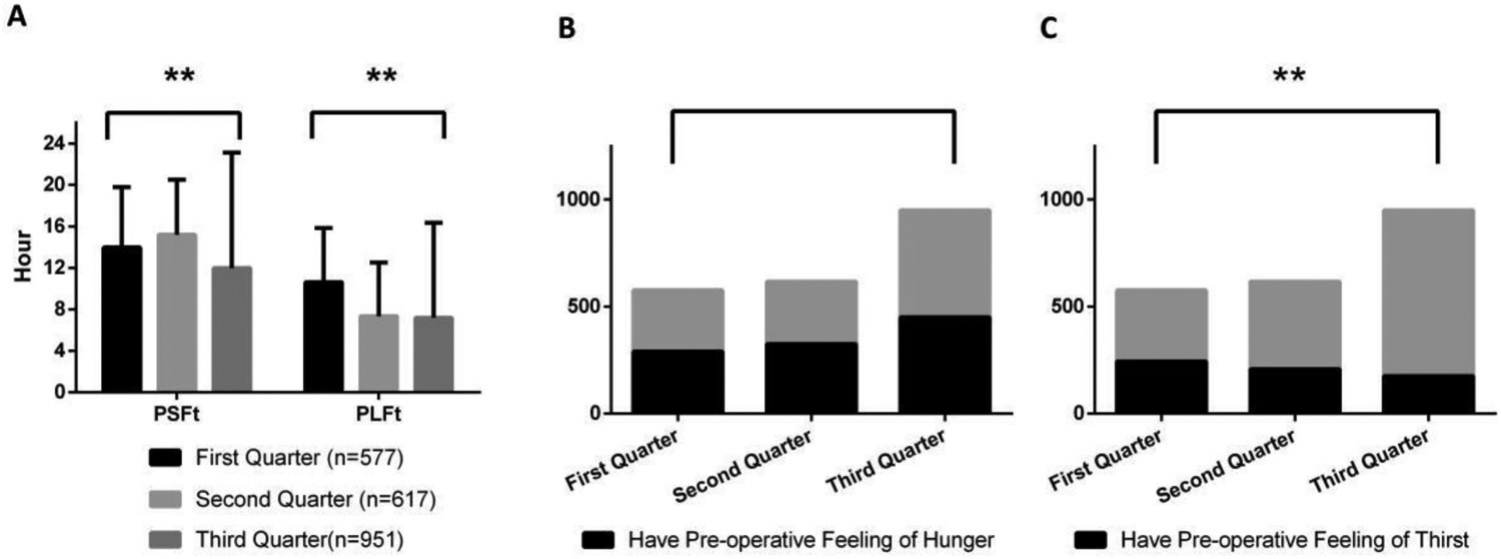
Department of anus & intestine surgery, **(A)** preoperative liquid fasting time (PLFt) and of preoperative solid fasting time (PSFt) of patients, **(B)** preoperative feeling of hunger (PFoH) of patients, **(C)** preoperative feeling of thirst (PFoT) of patients. **p ≤ 0.001.

**Figure 5:**
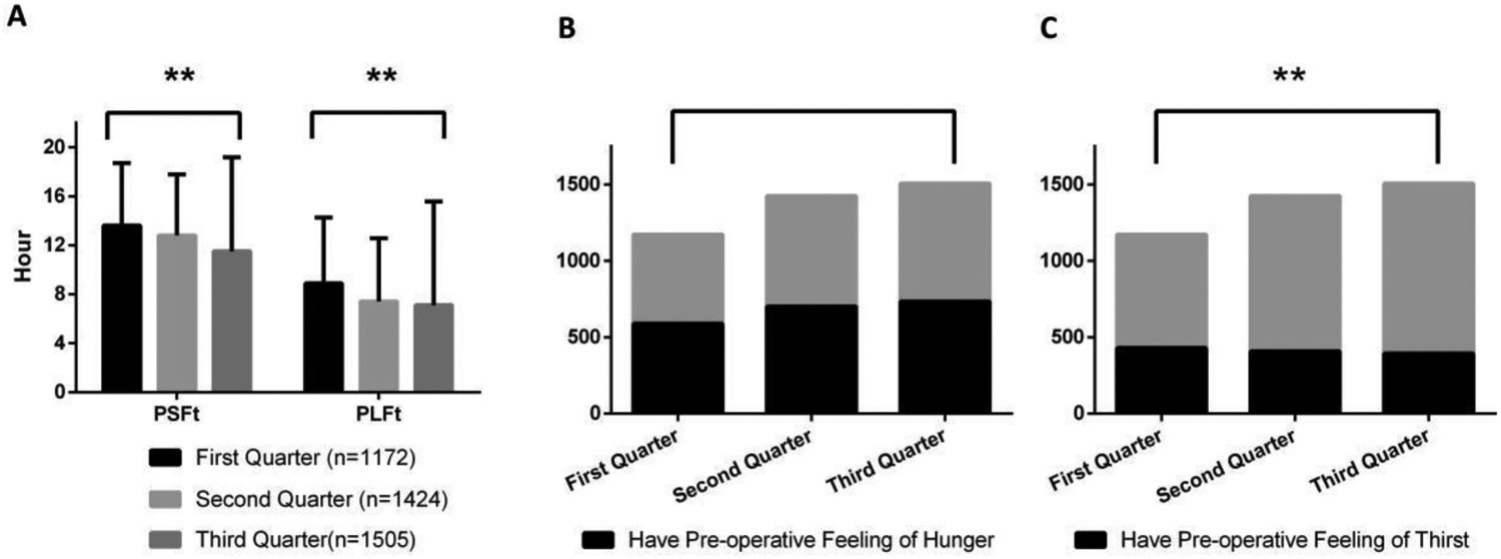
Department of hepatobiliary surgery, **(A)** preoperative liquid fasting time (PLFt) and of preoperative solid fasting time (PSFt) of patients, **(B)** preoperative feeling of hunger (PFoH) of patients, **(C)** preoperative feeling of thirst (PFoT) of patients. **p ≤ 0.001.

**Figure 6:**
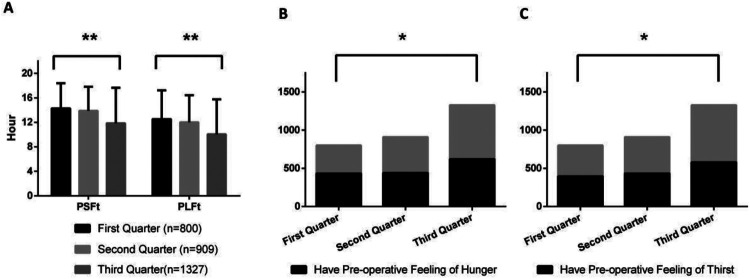
Department of ENT, **(A)** preoperative liquid fasting time (PLFt) and of preoperative solid fasting time (PSFt) of patients, **(B)** preoperative feeling of hunger (PFoH) of patients, **(C)** preoperative feeling of thirst (PFoT) of patients. **p ≤ 0.001, *p ≤ 0.05.

**Figure 7:**
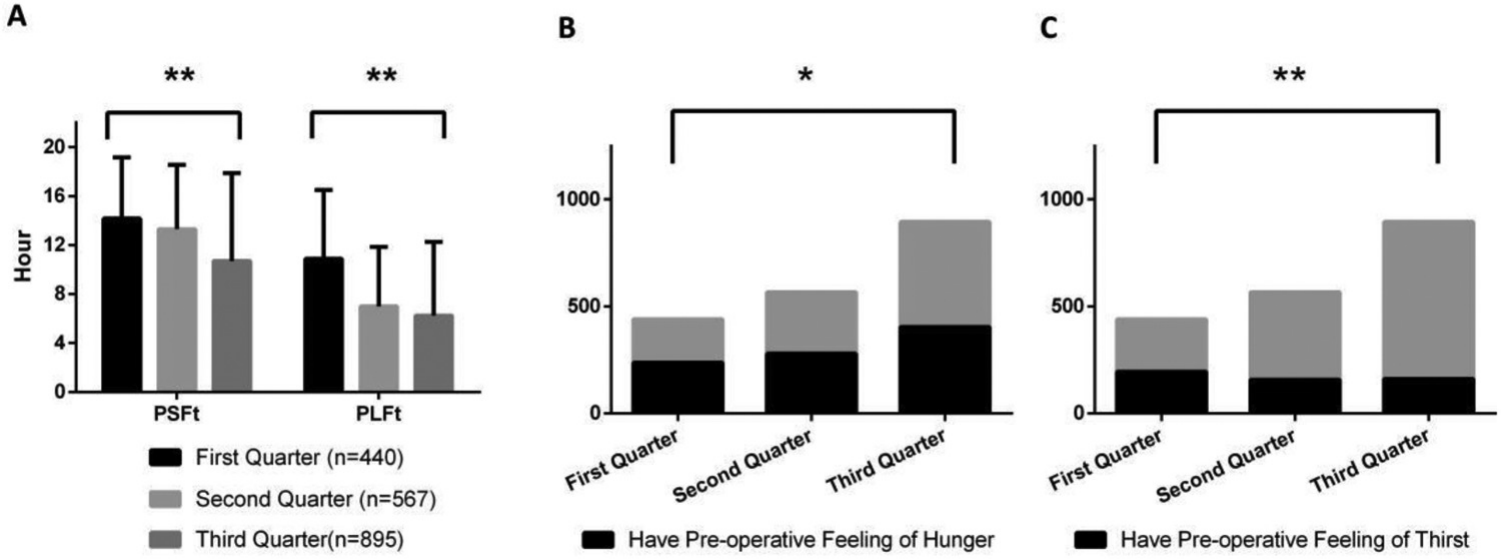
Department of thyroid & breast surgery, **(A)** preoperative liquid fasting time (PLFt) and of preoperative solid fasting time (PSFt) of patients, **(B)** preoperative feeling of hunger (PFoH) of patients, **(C)** preoperative feeling of thirst (PFoT) of patients. **p ≤ 0.001, *p ≤ 0.05.

**Figure 8:**
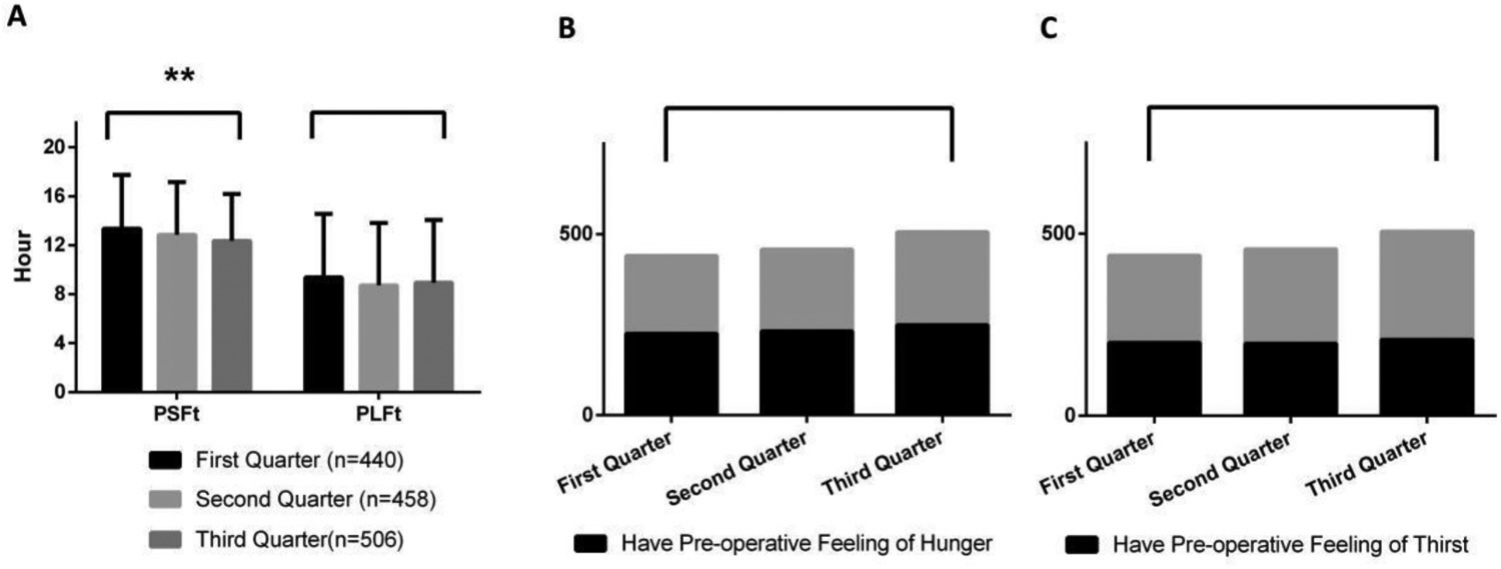
Department of cardiac & thoracic surgery, **(A)** preoperative liquid fasting time (PLFt) and of preoperative solid fasting time (PSFt) of patients, **(B)** preoperative feeling of hunger (PFoH) of patients, **(C)** preoperative feeling of thirst (PFoT) of patients. **p ≤ 0.001.

## Discussions

Our research findings indicated that the hospital-mediated comprehensive ERAS work significantly reduced preoperative fasting time in most departments. The PLFt in the Gynecology department decreased to 4.78 h, while in the Urology department, it decreased to 4.94 h. Multiple departments now have PSFt below or close to 10 h; however, there remains a gap in the fasting time recommended by the ERAS Association (8, 9).

This discrepancy is attributed to the practical challenges encountered in the hospital's operations. First, the first operation usually starts at about 8 a.m., and patients can drink clear liquids containing carbohydrates 2 h before the operation; however, it is impractical for patients to eat 4 h before the operation, which will disturb the normal sleep and physiological cycle of patients. Second, surgical patients may experience surgical sequence adjustments, including advanced or postponed surgery. This can result in surgery starting earlier or later than planned, which presents significant challenges to the precise fasting work. The strict adherence to fasting guidelines may compromise the smooth operation. Our current approach includes formulating a dietary schedule according to the surgical schedule and estimated duration and modifying it according to the specific situation on the day of surgery. ERAS protocols are generally well-established and relatively inflexible clinical procedures, and variations should not be substantial across countries. However, medical resources, patient profiles, perioperative management strategies, and health systems differ in every country, region, and medical institution. Therefore, the specific implementation of the ERAS concept in all medical institutions will vary greatly (16, 17).

Preoperative fasting is aimed at minimizing gastric contents to reduce the risk of aspiration and subsequent pulmonary aspiration, a rare but potentially life-threatening complication. In an analysis of aspiration events described by the American Society of Anesthesiologists in 2021, 57% of aspiration events resulted in death, while another 15% resulted in permanent severe damage. The literature suggests that consuming clear fluids containing carbohydrates up to 2 h before surgery is more beneficial than absolute fasting in terms of outcomes, with no evidence of increased risk (16). Following the recommendations of Enhanced Recovery After Surgery (ERAS) guidelines, we administered clear fluids containing carbohydrates to patients preoperatively, which the hospital provided to ensure safety and facilitate uniform management. Preoperative clear fluids containing carbohydrate intake can reduce the incidence of preoperative thirst and hunger, reduce insulin resistance, improve health, and possibly faster recovery (17). Additionally, clear fluids containing carbohydrates are easier to manage during the perioperative period than solid foods, including solid protein granules. Consequently, we have prioritized the improvements of protocols for clear fluids containing carbohydrates in our practice. Our statistical analysis indicates a significant reduction in PLFt in most departments. However, our results also indicate that the improvement in preoperative thirst incidence is more significant than the preoperative hunger rate. Therefore, it is necessary to investigate more appropriate and safer preoperative fasting protocols in future ERAS research.

Currently, the primary challenges facing the development and realization of the benefits of ERAS programs stem from two main factors. First, there is inadequate and inconsistent understanding and acceptance of ERAS knowledge among medical staff, including physicians, nurses, and anesthesiologists. Second, the successful implementation of ERAS programs requires multidisciplinary collaboration; therefore, it is unfeasible for a single department to conduct ERAS work comprehensively (9–11). Hospitals play a critical role in effectively mobilizing internal resources, including manpower and material resources, to address uneven distribution and inefficient utilization of resources within the hospital (18, 19). Hospital administrators, as leaders in the healthcare system (20), can assemble surgical, anesthesia, nursing, nutrition, and psychological personnel into an ERAS team, thereby promoting multidisciplinary cooperation and flexible combinations as the norm. Furthermore, hospitals can improve and standardize healthcare professionals' understanding of ERAS principles through regular educational activities. The implementation and benefits of ERAS programs can be significantly impacted through hospital-mediated implementation. Additionally, our research confirms that comprehensive advancement of ERAS programs under hospital guidance can effectively reduce preoperative fasting durations across departments and decrease the incidence of patient preoperative hunger and thirst. This is an important progress in our hospital's ERAS work. We have developed an ERAS work plan suitable for our hospital's management model, including the core team and ERAS pathway. We will share our experience with other ERAS projects in future work to achieve higher-level ERAS goals, thereby achieving the objective of shortening patient hospitalization time, reducing complications, and reducing medical expenses.

This study still has several limitations. First, the research only observed changes in patients' preoperative experiences after implementing the fasting and feeding ban program. However, the true goal of ERAS is to shorten hospital stays and achieve better outcomes. Consequently, in future work, it is necessary to establish a more comprehensive information data collection system to obtain more data relevant to patient benefits. Furthermore, the ERAS pathway involves multiple work projects, and for the comprehensive promotion of ERAS work reform in our hospital, the fasting and feeding ban program was implemented as the initial work objective. Additionally, our results demonstrate the feasibility of hospitals as the primary implementers of ERAS benefits. Subsequently, based on our work experience, we will conduct work at multiple levels covering preoperative, intraoperative, and postoperative stages to benefit patients. Lastly, the long-term benefits of implementing the ERAS program are unclear. Therefore, in future work, it is imperative to conduct longer follow-up studies to clarify the progress of ERAS-related work and evaluate the potential for long-term benefits.

In conclusion, implementing hospital-mediated ERAS is feasible and can effectively mobilize resources across departments, coordinate efforts, and address challenges in ERAS implementation. However, the long-term benefits of hospital-mediated ERAS initiatives require comprehensive ERAS protocols and longer follow-up studies for clarity. Additionally, the ERAS procedures need further improvements.

## Data Availability

The raw data supporting the conclusions of this article will be made available by the authors, without undue reservation.
